# Diverse *Francisella tularensis* Strains and Oropharyngeal Tularemia, Turkey

**DOI:** 10.3201/eid2101.141087

**Published:** 2015-01

**Authors:** Yasemin Özsürekci, Dawn N. Birdsell, Melda Çelik, Eda Karadağ-Öncel, Anders Johansson, Mats Forsman, Amy J. Vogler, Paul Keim, Mehmet Ceyhan, David M. Wagner

**Affiliations:** Author affiliations: Hacettepe University, Ankara, Turkey (Y. Özsürekci, M. Çelik, E. Karadağ-Öncel, M. Ceyhan);; Northern Arizona University, Flagstaff, Arizona, USA (D.N. Birdsell, A.J. Vogler, P. Keim, D.M. Wagner);; Umeå University, Umeå, Sweden (A. Johansson);; Swedish Defence Research Agency, Umeå (M. Forsman);; Translational Genomics Research Institute, Flagstaff (P. Keim)

**Keywords:** *Francisella tularensis*, tularemia, Turkey, SNP, canSNP, clinical sample, bacteria

**To the Editor:** Tularemia is a zoonosis caused by the bacterium *Francisella tularensis*; the main forms of disease that occur in humans are ulceroglandular/glandular, oculoglandular, oropharyngeal, and respiratory. In Turkey, tularemia outbreaks were described as early as 1936–1938 (*1*), but tularemia was not reportable until 2004. Recently, multiple tularemia outbreaks in Turkey have been described, including in regions where the disease has not been previously reported; it is now considered a reemerging zoonotic disease in Turkey (*1*).

The only *F. tularensis* subspecies found in most of Eurasia, including Turkey, is *holarctica*. Genetic diversity is low, probably because emergence is recent (*2*). However, discovery of whole-genome single-nucleotide polymorphisms (SNPs), coupled with subsequent canonical SNP (canSNP) analyses, have identified numerous phylogenetic groups within this subspecies. The distinct phylogeographic patterns provide insight into its evolutionary history (*3*–*7*).

From December 2009 through January 2011, tularemia outbreaks increased in Turkey, primarily in the central region (*8*). Oropharyngeal tularemia was diagnosed for 14 patients (13 children, 1 adult), and fine-needle lymph node aspiration was performed at the Pediatric Infectious Diseases Unit at Hacettepe University, Ankara. DNA was extracted from these 14 samples (QIAamp DNA Mini Kit; QIAGEN, Hilden, Germany) and screened by using a PCR selective for the *tul4* gene region specific to *F. tularensis* (*9*); all 14 samples were positive for *F. tularensis* (Table). Residual, de-identified portions of these 14 DNA extracts were used for this study.

**Table:** *Francisella tularensis*–positive clinical samples from 14 patients with oropharyngeal tularemia, Turkey, December 2009–January 2011*

Patient no. (sample no.)	City	CanSNP subgroup†	MLVA genotype‡
1 (F0737)	Corum	B.20/21/33	i
2 (F0738)	Cankırı	B.28/29	b
3 (F0739)	Yozgat	B.28/29	b
4 (F0740)	Zonguldak	B.7/8	a
5 (F0741)	Corum	B.20/21/33	e
6 (F0742)	Corum	B.20/21/33	e
7 (F0743)	Corum	B.20/21/33§	ND
8 (F0744)¶	Bala/Ankara	B.20/21/33	e
9 (F0745)	Ankara	B.20/21/33	d
10 (F0746)	Corum	B.20/21/33	j
11 (F0747)	Bala/Ankara	B.20/21/33	g
12 (F0748)	Corum	B.20/21/33	f
13 (F0749)	Ankara	B.20/21/33	c
14 (F0750)	Emirdağ/Afyon	B.20/21/33	h

*Clinical samples collected in 2011 from patients with cervical lymphadenitis at Hacettepe University Medical Faculty, Pediatric Infectious Disease unit, Ankara, Turkey. CanSNP, canonical single-nucleotide polymorphism; MLVA, multilocus variable-number tandem-repeat analysis; ND, not determined. †Subgroups published in (*3**,**4**,**6**,**7*). CanSNP branches tested on samples in this study: B.3, B.4, B.5, B.6, B.7, B.8, B.9, B.10, B.11, B.13, B.20, B.21, B.22, B.26, B.27, B.28, B.29, B.30, B.31, B.32, and B.33. ‡MLVA markers (M03, M05, M06, M20) (*2*). §Subgroup classification based on approximation because genotype for B.33 remains unresolved. ¶This patient was an adult (all others were children).

Genetic characterization led to assignment of these 14 samples to multiple phylogenetic groups within *F. tularensis* subsp. *holarctica*. Analysis with 18 previously described (*3*,*4*,*6*,*7*) canSNP assays (Table) led to assignment of the 14 samples to 3 previously described phylogenetic groups within this subspecies: B.20/21/33 (n = 11), B.28/29 (n = 2), and B.7/8 (n = 1) (Table; Technical Appendix). To identify additional genetic diversity, we used 4 previously described (*2*) variable-number tandem-repeat markers (M03, M05, M06, and M20) and identified 10 genotypes among the 14 samples, 8 of which were identified in the 11 B.20/21/33 samples (Table). 

The genetic diversity among these samples and their widespread geographic origins from 6 provinces in central Turkey (Table; Technical Appendix) suggest that the patients contracted tularemia from multiple independent sources. These sources might have been contaminated drinking water, which has been implicated as the source of human tularemia in previous outbreaks in Turkey (*1*) and could account for oropharyngeal tularemia in the 14 patients reported here.

The finding of these 3 phylogenetic groups within Turkey expands the known geographic range of these phylogenetic groups within *F.* *tularensis* subsp. *holarctica*. The presence of group B.28/29 *F.* *tularensis* in Turkey is not surprising; isolates belonging to this group were previously identified in bordering Georgia (*3*). Likewise, the presence of group B.20/21/33 *F.* *tularensis* is not unexpected, given the wide geographic distribution (Sweden, Finland, Russia, and Hungary [*4*]) of organisms belonging to this group (Technical Appendix). Isolating group B.7/8 *F.* *tularensis,* previously thought to occur only in Scandinavia, in Turkey is of particular interest, given the relatively basal position of this group in the *F. tularensis* subsp. *holarctica* phylogeny (Technical Appendix). Indeed, descendants of this group have, to date, been identified from North America only, suggesting a transfer from the Old World to the New World within this lineage (*7*). The circumstances of this transfer are unknown but might be discerned through additional knowledge of the geographic extent and genetic diversity of organisms in the B.7/8 group.

It has been suggested that Scandinavia might be the source of the historical spread of tularemia to the rest of Europe and might be the origin of the ancestor to the B.13 clade (*5*). This suggestion was previously argued because *F. tularensis* subsp. *holarctica* isolates from Sweden have yielded more phylogenetic diversity than isolates from any other country. Indeed, except for the B.27 clade, much of the known phylogenetic diversity of this organism within Europe is present in Sweden (*5*). Some of the largest sets of analyzed samples originated in Sweden (*5*–*7*), whereas eastern Europe and much of Asia remain mostly undersampled. The high genetic diversity identified in our very limited sample set from Turkey is notable and includes 2 major lineages (B.7 and B.13; Technical Appendix). These findings, together with the recent discovery that organisms of multiple *F. tularensis* subsp. *holarctica* phylogenetic groups exist in China (*10*), suggest that much additional phylogenetic diversity within this subspecies remains to be discovered in Eurasia, which will provide better information about the evolutionary history and historical spread of *F. tularensis* subsp. *holarctica*.

We have demonstrated that high-resolution genetic characterization of *F. tularensis* DNA extracted from biopsy samples is possible, and we conclude that oropharyngeal human tularemia in Turkey is caused by organisms of multiple distinct phylogenetic groups within this subspecies. This pattern, together with the wide geographic distribution of the 14 patients within Turkey (Technical Appendix), suggests that the persons infected by *F. tularensis* during the 2009–2011 outbreaks in Turkey obtained their infections from multiple environmental sources.

Technical Appendix Global phylogeography of *Francisella tularensis* subsp. *holarctica* and location of 14 clinical samples from Turkey.
