# Supplementary material for: Diverse Francisella tularensis Strains and Oropharyngeal Tularemia, Turkey
Source: Emerg Infect Dis. 2015 Jan;21(1):173–5. doi: 10.3201/eid2101.141087 (PMC4285279; doi:10.3201/eid2101.141087)
Supplement: Technical Appendix — Global phylogeography of Francisella tularensis subsp. holarctica and location of 14 clinical samples from Turkey. [file 14-1087-Techapp-s1.pdf]

DOI: http://dx.doi.org/10.3201/eid2101.141087

Technical Appendix

Diverse *Francisella tularensis* Strains and Oropharyngeal Tularemia, Turkey

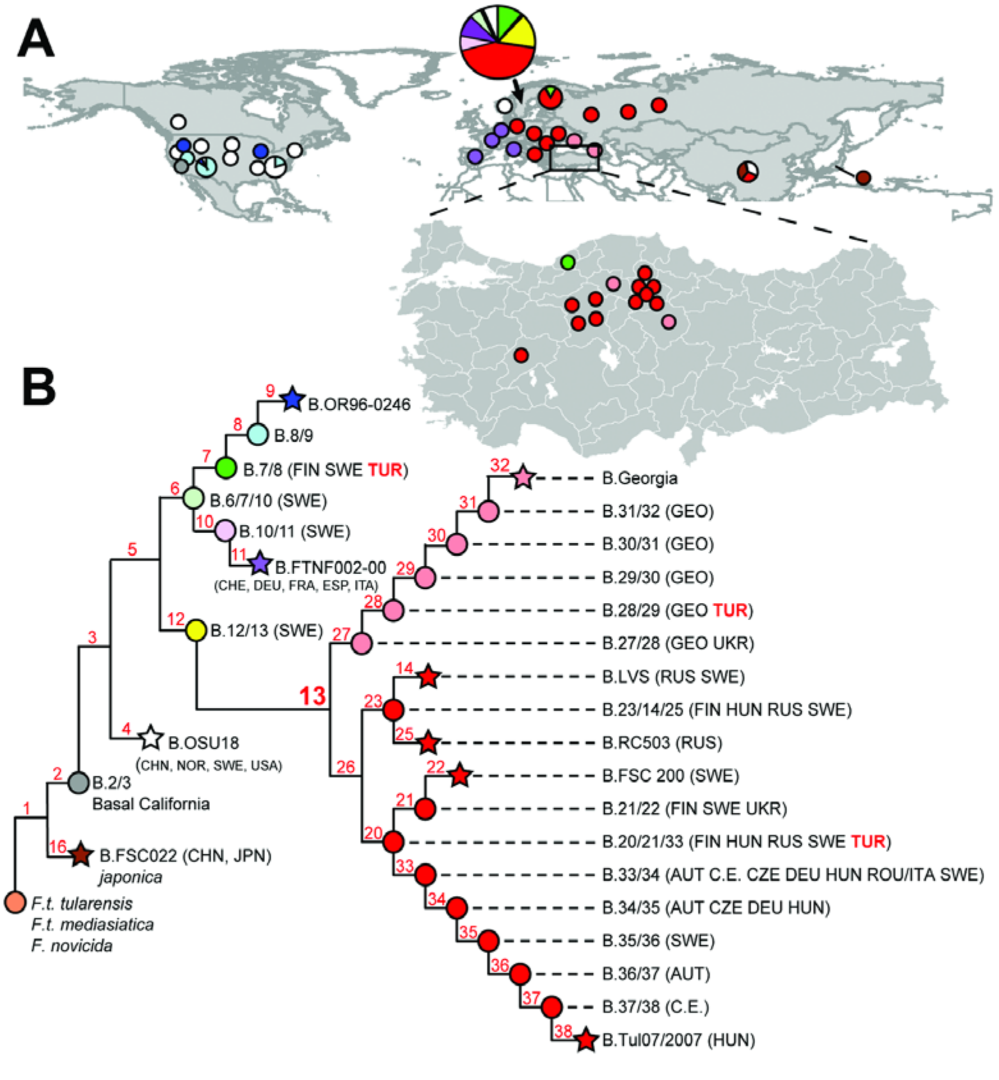

Appendix Figure. Global phylogeography of *Francisella tularensis* subsp. *holarctica* and location of 14 clinical samples from Turkey. A) Global distribution of known phylogenetic groups (*3*,*4*,*6*,*7*) and expanded map of Turkey indicating provinces where the 14 patients lived (colored circles). Circle colors indicate phylogenetic groups to which the 14 samples were assigned. B) Current single-nucleotide polymorphism–based phylogenetic tree for *F. tularensis*. Red numbers indicate canonical single-nucleotide

polymorphisms (*3*,*4*,*6*,*7*). Countries of origin for strains assigned to relevant phylogenetic groups are indicated as follows: AUT, Austria; C.E., central Europe, unknown country; CZE, Czech Republic; DEU, Germany; FIN, Finland; GEO, Georgia; HUN, Hungary; ITA, Italy; ROU, Romania; RUS, Russia; SWE, Sweden; TUR, Turkey; UKR, Ukraine). Bold red text indicates phylogenetic placement of the 14 clinical samples examined in this study.
